# Differential Transcriptional Regulation in Roots of Tomato Near-Isogenic Lines in Response to Rapid-Onset Water Stress

**DOI:** 10.3389/fpls.2017.00166

**Published:** 2017-02-21

**Authors:** Erin M. Arms, Zhanghang Yan, Dina A. St.Clair

**Affiliations:** ^1^St. Clair Lab, Plant Sciences Department, University of California DavisDavis, CA, USA; ^2^Korf Lab, Genome Center, University of California DavisDavis, CA, USA

**Keywords:** tomato, transcriptional regulation, water-stress, mRNA-Seq, root chilling, abiotic stress

## Abstract

Cultivated tomato (*Solanum lycopersicum* L.) is susceptible to abiotic stresses, including drought and chilling stress, while its wild relative (*Solanum habrochaites*) exhibits tolerance to many abiotic stresses. Chilling roots to 6°C induces rapid-onset water stress by impeding water movement from roots to shoots. Wild *S. habrochaites* responds to root chilling by closing stomata and maintaining shoot turgor, while cultivated tomato fails to close stomata and wilts. This phenotypic response (shoot turgor maintenance under root chilling) is controlled by a major QTL *stm9* on chromosome 9 from *S. habrochaites* that was previously high-resolution mapped to a 0.32 cM region, but its effects on transcriptional regulation were unknown. Here we used paired near isogenic lines (NILs) differing only for the presence or absence of the *S. habrochaites* introgression containing *stm9* in an otherwise *S. lycopersicum* background to investigate global transcriptional regulation in response to rapid-onset water stress induced by root chilling. NIL175 contains the *S. habrochaites* introgression and exhibits tolerance to root chilling stress, while NIL163 does not contain the introgression and is susceptible. RNA from roots of the two NILs was obtained at five time points during exposure to root chilling and mRNA-Seq performed. Differential expression analysis and hierarchical clustering of transcript levels were used to determine patterns of and changes in mRNA levels. Our results show that the transcriptional response of roots exposed to chilling stress is complex, with both overlapping and unique responses in tolerant and susceptible lines. In general, susceptible NIL 163 had a more complex transcriptional response to root chilling, while NIL175 exhibited a more targeted response to the imposed stress. Our evidence suggests that both the tolerant and susceptible NILs may be primed for response to root-chilling, with many of these response genes located on chromosome 9. Furthermore, serine/threonine kinase activity likely has an important role in the root chilling response of tolerant NIL175.

## Introduction

Plant exposure to abiotic stresses may limit plant growth and development, and in crop plants may ultimately affect yield. Abiotic stresses such as drought, salinity, and temperature extremes may lead to turgor loss, disorganization of membranes, loss of protein activity, increased levels of reactive oxygen species (ROS) and subsequent oxidative damage (Krasensky and Jonak, 2012). Domesticated crop plants are often sensitive to abiotic stresses, while their related wild species may exhibit tolerance. For tropical and subtropical species, exposure to chilling temperatures (above 0°C and below 10°C) can be especially damaging (Geisenberg and Stewart, 1986; Allen and Ort, 2001). Exposure of roots to chilling temperatures inhibits hydraulic conductance, which results in reduced water uptake, leading to rapid-onset water stress during the course of a few hours or days if the plant is not able to respond by closing its stomata (Wilson, 1976; Aroca et al., 2001, 2012; Bloom et al., 2004). Seedlings exposed to cold soils during the spring may experience root chilling stress leading to wilting and injury due to decreased water and nutrient uptake that affects plant growth and yield (Nobel, 1983, 1999; Allen and Ort, 2001).

Cultivated tomato (*Solanum lycopersicum* L.) was domesticated from the wild cherry tomato which is native to mesic, tropical environments (Rick, 1976, 1983, 1988; Bergougnoux, 2014). Most wild tomato species have tolerance to various abiotic stresses. Wild tomato, *Solanum habrochaites* S. Knapp and D.M. Spooner, grows in the Peruvian Andes at altitudes up to 3300 m and thrives in xeric habitats at chilling temperatures detrimental to *S. lycopersicum* (Dalziel and Breidenbach, 1982; Wolf et al., 1986; Vallejos and Pearcy, 1987; Jung et al., 1998; Venema et al., 1999). Bloom et al. (2004) reported that *S. habrochaites* responds to rapid-onset water stress induced by root chilling by closing its stomata, thereby maintaining water potential and shoot turgor, while stomata of *S. lycopersicum* remain open, resulting in wilting, and eventual tissue damage.

Previously, a major effect QTL controlling shoot turgor maintenance under root chilling was identified in a *S. lycopersicum* cv. T5 × *S. habrochaites* acc. LA1778 backcross population (Truco et al., 2000). This QTL (designated *stm9*) was recently high-resolution mapped to a 0.32 cM region on the short arm of chromosome 9 from *S. habrochaites* (Arms et al., 2015). As part of this effort, we developed a pair of near isogenic lines (paired NILs) for chromosome 9. NIL175 (root-chilling tolerant) contains a chromosome 9 introgression from *S. habrochaites* LA1778 spanning markers T1670 to T0532 (including *stm9*) in an otherwise *S. lycopersicum* cv. T5 background, while NIL163 (root-chilling susceptible) does not contain any introgressions from *S. habrochaites* LA1778. These NILs are genetically identical except for the presence or absence of the chromosome 9 introgression, and were used in the present study to analyze transcriptional responses to root chilling (as described below).

To explore the basis of the differential response of *S. lycopersicum* and *S. habrochaites* to chilling, Bloom et al. (2004) conducted root chilling experiments with grafted tomato plants consisting of differing genotypes for root and shoot. Grafted plants with shoots of a wilting genotype (*S. lycopersicum*) showed tolerance to root chilling when grafted onto roots of a non-wilting genotype (containing a chromosome 9 introgression from *S. habrochaites* acc. LA1778) indicating that the root of the non-wilting genotype prevented shoot wilting. Reciprocal grafts (i.e., shoot of a non-wilting genotype grafted to a root of a wilting genotype) responded similarly to a wilting genotype, indicating that the root of the wilting genotype was unable to trigger stomatal closure upon exposure to chilling (Bloom et al., 2004). Current evidence suggests that the basis for this differential response is root-to-shoot signaling that controls stomatal closure (Bloom et al., 2004; Easlon et al., 2013). Recent work by Easlon et al. (2013) demonstrated that shoot turgor maintenance under root chilling is a trait shared by some other wild tomato species, and is controlled by a region on the short arm of chromosome 9 that is syntenic to that containing QTL *stm9*. Collectively, the results from Arms et al. (2015), Bloom et al. (2004), and Easlon et al. (2013) suggest that when roots are exposed to chilling, tolerant genotypes transmit a signal from roots to shoots that results in stomatal closure, thereby preventing shoot wilting. Furthermore, the gene(s) responsible for the signal are likely non-functional and/or absent in cultivated tomato. Although the identity of the signal is unknown, it is possible that several parallel signaling pathways are operating, and these may involve abscisic acid (ABA), antioxidants, and/or primary elements (such as calcium or potassium; Smallwood and Bowles, 2002; Ramachandra Reddy et al., 2004). An understanding of the underlying transcriptional response to chilling in roots may help reveal the nature of this signal.

Plant transcriptional responses to abiotic stresses are often complex, involving a core set of multi-stress response genes, and overlap in the gene pathways involved in stress responses (Walley and Dehesh, 2010). A number of methods can be used to study transcriptional regulation. Sequencing of mRNA (mRNA-Seq) provides transcript read data (transcriptome) for samples of interest. Once obtained, transcriptome data may be analyzed with different methods including differential expression, cluster, and gene ontology enrichment analyses. Differential expression analysis is used to identify genes that have significantly different levels of transcription (known as differentially expressed or DE genes) and involves the statistical comparison of RNA sequencing datasets (Anders and Huber, 2010). Cluster analysis identifies groups of genes that share the same pattern of transcriptional regulation across a series of conditions and/or time-points (Fraley et al., 2012). Gene ontology (GO) enrichment analysis identifies statistically significantly enriched GO terms from a list of genes identified by differential expression or cluster analysis (Ashburner et al., 2000). Significant GO terms help elucidate the gene families or networks that are over-represented in the gene list, helping to reveal the biological processes affected by a given set of conditions (Khatri and Draghici, 2005; Reimand et al., 2007).

We hypothesized that the QTL *stm9* region from *S. habrochaites* that controls the plant phenotype of shoot turgor maintenance under root chilling stress also has a significant effect on genome-wide transcriptional regulation in roots exposed to chilling. To test this hypothesis, we subjected our paired NILs, NIL175 (root-chilling tolerant) and NIL163 (root-chilling susceptible), to a time-course experiment under root chilling conditions. We used mRNA-Seq to obtain root transcriptome data for the two NILs, and conducted analyses of differential gene expression, clustering, and gene ontology. Our primary aims were to: identify differentially expressed genes, gene families, and gene networks involved in the responses of NIL175 and NIL163 to rapid-onset water stress induced by root chilling; and identify genome-wide transcriptional patterns involved in root responses to chilling.

## Materials and methods

### Plant material and growth conditions

We used two near isogenic lines (paired NILs) that were genetically identical, differing only for the presence or absence of a chromosome 9 introgression from *S. habrochaites* acc. LA1778 in an otherwise *S. lycopersicum* cv. T5 genetic background. Development of the NILs (designated NIL175 and NIL163) and their graphical genotypes are described in Easlon et al. (2013). Briefly, NIL175 contains a *S. habrochaites* introgression on chromosome 9 spanning markers T1670 to T0532, an interval which includes QTL *stm9*, and this NIL maintains shoot turgor (i.e., exhibits tolerance) when exposed to root chilling. NIL163 does not contain any introgressions from *S. habrochaites* and is chilling sensitive, exhibiting shoot wilting when its roots are exposed to chilling stress (Easlon et al., 2013).

All plant flats and hydroponic tanks used in our experiments were located in a greenhouse at UC Davis in Davis, California, with ambient daytime conditions of 25–37°C and 55–80% relative humidity, and nighttime conditions of 18–25°C and 20–55% relative humidity. Seeds of NIL175 and NIL163 were seeded into 72-cell flats containing SuperSoil potting media (Rod McLellan Co.), watered daily, and fertilized with a 10:30:20 NPK solution 10 days after planting. After 2 weeks, roots of seedlings of NIL175 and NIL163 were carefully washed free of potting media in deionized water and transferred to a hydroponic growth tank containing 20% strength modified Hoagland's solution (Epstein and Bloom, 2005). Plants were maintained in the tank containing the aerated, circulating root solution at 20°C under ambient illumination. After 1 week, plants were transferred and randomized (see Section Experimental Design and Sample Collection) in a separate refrigerated hydroponic tank at 20°C containing fresh 20% strength modified Hoagland's solution. Plants were acclimated overnight prior to initiation of the root chilling experiment the next day.

### Experimental design and sample collection

A completely randomized design was employed for conducting a time-course experiment, with two genotypes (NIL175 and NIL163), five sampling time-points (0, 1, 1.5, 2, and 4 h from initiation; see description below), and three biological replicates per time-point/genotype combination. Experimental conditions were similar to those used in our prior experiments on shoot turgor maintenance under root chilling (Truco et al., 2000; Goodstal et al., 2005; Arms et al., 2015). At the initiation of the experiment, the temperature setting on the hydroponic tank was decreased from 20 to 6°C, and a supplemental cooling wand was placed in the solution to aid chilling until the target temperature of 6°C was reached at 2 h. At each of the five time-points, three plants of each NIL were collected (for a total of 30 samples). A table describing each sample is available (see Supplementary Table 1). Plants were removed from the hydroponic tank, roots briefly and quickly dried on absorbent paper towels, and whole plants placed in pre-labeled and autoclaved aluminum foil envelopes before being flash frozen in liquid nitrogen. Samples were transported to the lab in liquid nitrogen, transferred to a −80°C freezer, and stored at −80°C until processed for mRNA extraction (see Section Library Preparation and Sequencing). Several additional plants of NIL175 and NIL163 were also included in the experiment to observe and confirm the expected phenotypes at 4 h. Those are: shoot wilting in NIL163 and maintenance of shoot turgor in NIL175 (Arms et al., 2015).

The five time-points were selected to capture biologically important time-points in the plant stress response to root chilling, including the transition from no stress (above 10°C) and the start of chilling stress (below 10°C; Geisenberg and Stewart, 1986; Allen and Ort, 2001). At 0 h, the plants were at ambient greenhouse temperatures with a root solution temperature of 20°C. Plants collected at 0 h served as non-stressed controls for all subsequent time-points. It should be noted that changes in other environmental conditions such as light quantity and quality during the course of an experiment performed over time may affect transcriptional regulation of some genes. At 1 h, the root solution decreased to ~11–12°C, just above the 10°C threshold for root chilling stress. At 1.5 h, the root solution decreased to ~8–9°C, below the 10°C threshold for root chilling stress. At 2 h, the root solution reached 6°C, where it was maintained for the duration of the experiment. Susceptible NIL163 showed first signs of wilt (loss of turgor in leaf tips) at 2 h. At 4 h, NIL163 was fully wilted, while NIL175 maintained shoot turgor. In our prior experiments, phenotyping for shoot turgor maintenance was performed at 4 h (Truco et al., 2000; Goodstal et al., 2005; Arms et al., 2015). After sample collection and phenotyping at 4 h, the experiment was terminated. For a more in depth description of phenotypic responses of the NILs and experimental conditions, we refer the reader to Arms et al. (2015).

### mRNA library preparation and sequencing

Approximately one quarter of the root mass of each sample (30 in total) was ground in liquid nitrogen to a fine powder with a mortar and pestle. Following pulverization, 100 mg of each sample was processed using the Qiagen RNeasy mini kit (www.qiagen.com). Isolated RNA was quantified using Life Technologies Qubit RNA Broad Range Assay kit (www.lifetechnologies.com), and quality control was performed with the Agilent Bioanalyzer RNA 6000 Nano kit (www.agilent.com). Messenger RNA (mRNA) from each sample was isolated from 10 μg of total RNA as input using the Bioo Scientific NEXTflex Poly(A) Beads isolation kit (www.biooscientific.com). Isolated mRNA was quantified using the Life Technologies Qubit RNA High Sensitivity Assay kit (www.lifetechnologies.com).

Sequencing mRNA libraries were prepared for each sample using the Bioo Scientific NEXTflex Rapid RNA-Seq kit with NEXTflex RNA-Seq barcodes (1–30 were used from the 48 barcode set; www.biooscientific.com; see Supplementary Table 1). Library quality control was performed on the Agilent Bioanalyzer with the DNA High Sensitivity DNA kit (www.agilent.com), and quantified via qPCR at the UC Berkeley Vincent J. Coates Genomics Sequencing Laboratory (GSL) (http://qb3.berkeley.edu/gsl/). All barcoded libraries were pooled and sequenced at the GSL on 12 lanes of single-end 50 sequencing on the Illumina Hi-Seq 2000 (www.illumina.com). The barcodes enabled sorting of the resulting reads from each sequencing lane with CASSAVA v1.8.2 (www.illumina.com) at the GSL before further analysis.

### Analysis of differential transcript expression

All read data were uploaded to and analyzed on the iPlant Discovery Environment using supported bioinformatics tools (https://de.cyverse.org/de/). For each of the 30 samples, all reads were concatenated into a single file, and quality control was performed. Scythe adapter-trimming was used to remove 3′-end adapter contamination, and Sickle quality-based-trimming was used to remove low quality reads. Read mapping statistics are provided (see Supplementary Table 1; Buffalo, 2010; Joshi and Fass, 2011). FastQC 0.10.1 (multi-file) was then used to check the quality of the read collection. Reads from each library were aligned to the *S. lycopersicum* cv. Heinz 1706 reference genome (SL2.50; solgenomics.net) with GSNAP (Genomic Short-read Nucleotide Alignment Program) using default settings and the quality format option Sanger (Supplementary Table 1; Wu and Nacu, 2010). GSNAP output SAM files were converted to BAM files using SAM-to-BAM, and then BAM-to-Counts was used to quantify the number of reads mapping to each *S. lycopersicum* ITAG v2.40 annotated transcript in FPKM (fragments per kilobase of transcript per million mapped reads). Number of reads mapped to each transcript per sample are available in Supplementary Table 2. To identify differentially expressed (DE) transcripts (genes), pairwise comparisons of different time-point/NIL combinations were made using DEseq1.0, with a *P* = 0.05 significance threshold. Pairwise comparisons were done between NILs at each time-point, as well as between the 0 h control and each subsequent time-point within each NIL. DEseq controls for false discovery rate due to multiple testing using the Benjamini-Hochberg procedure. All significantly DE genes were identified using this corrected *P*-value (Anders and Huber, 2010).

### Data availability

The datasets supporting the conclusions of this article are included within the article and its supplementary files. The raw Illumina sequences are publicly available via the NCBI Sequence Read Archive as of January 1st, 2017, under the following accession number: SRP081139.

### Transcript expression pattern clustering

Clustering of transcript expression patterns across the five time-points for NIL175 and NIL163 was performed using Mclust v5.0.1 (www.stat.washington.edu/mclust/) in R64 v3.2.0 (www.r-project.org/). Mclust uses a mixed method that combines model-based hierarchical clustering, EM for Gaussian mixture models, and BIC to optimize clustering results, including number of clusters (Fraley et al., 2012). The log average of FPKM values across the three biological replicates of a NIL at each time-point for each gene was used as input. After Mclust analysis, trend lines for each cluster were produced by averaging the values for all genes in that cluster at each time-point. Within each NIL, trend lines were grouped according to transcription pattern to produce “Trend Groups,” each of which included all individual clusters with the same relative transcriptional pattern regardless of the level of transcription. Trend Groups may therefore represent more than one cluster, but each cluster will only be included in a single Trend Group. The cluster and Trend Group assignment for each gene (for both NIL175 and NIL163) are included in a Supplementary File (Supplementary Table 2). Trend Groups were then compared between the two NILs. Trend Groups identified in both NIL175 and NIL163 were denoted with capital letters A–M, and Trend Groups identified in a single NIL were indicated with lower case letters n–v. Trend Group A consisted of a single cluster for each NIL, which represented no detectable level of transcript at any time-point (i.e., FPKM = 0).

### Venn visualization and GO enrichment analysis

Results of the differential expression and Trend Group analyses were visualized via area-proportional Venn diagrams using BioVenn (www.cmbi.ru.nl/cdd/biovenn/). Visualizations and comparisons between NIL175 and NIL163 for DE genes identified in time-point comparisons to the 0 h control were used to identify overlapping and unique DE genes. BioVenn was also used to visualize and compare gene lists from NIL175 and NIL163 of Trend Groups A–M. Trend Groups (n–v) unique to either NIL175 or NIL163 were not included in Venn diagram visualization. Output lists of unique and overlapping genes from Venn diagram analyses were used as input gene lists for GO enrichment analysis using g:Profiler vr1435_e80_eg27 (biit.cs.ut.ee/gprofiler/). *S. lycopersicum* ITAG v2.40 was specified, and default settings were used, with the exception of a user-defined *P*-value of 0.05 to return only statistically significant results (used to produce tables provided as supplementary files due to size). The default g:SCS (Set Counts and Sizes) threshold method was used to control for false positives (inflated type I error) from multiple testing. This correction method takes into account the effects of multiple tests and GO terms that are not independent, but are a hierarchically organized collection of related general and specific terms (Reimand et al., 2007). Gene lists that returned significantly enriched GO terms were rerun using the “Best per parent group” option, where “parent” was defined as the highest-level GO term in a hierarchy of related GO terms (used to produce in-text tables). This setting returned results that grouped related GO terms and represented them with the most statistically significant parent GO term to aid in parsing of the data.

## Results

### Differential transcript expression analysis of NIL175 and NIL163

Differential expression analysis identified DE genes for all time-point/NIL combinations, with generally increasing numbers of DE genes as the experiment progressed. A list of differentially expressed genes for all time-point/NIL combinations is available in a table (see Supplementary Table 3). In comparisons between the NILs at each of five time-points, the number of DE genes ranged from 20 at 0 h to 63 at 4 h. Chromosome 9 had the greatest number of DE genes, ranging from 13 at 0 h to 22 at 2 h. Interestingly, DE genes located across all 12 chromosomes were identified only at 2 h (Supplementary Table 3).

**Figure 1:**
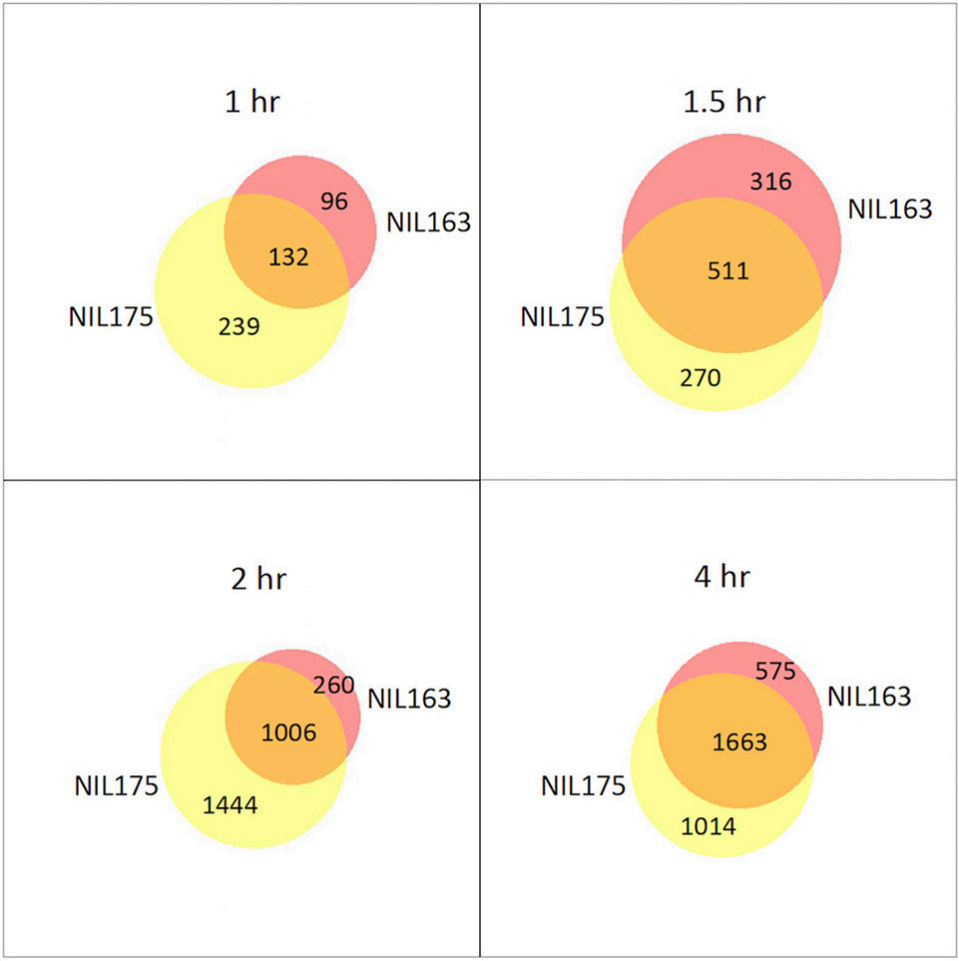
**Differentially expressed genes between NIL175 and NIL163 at four time-points**. Venn diagrams showing number of unique genes from DE analysis for NIL175 (yellow), NIL163 (pink) and for overlapping genes (orange). Diagrams were created using BioVenn (http://www.cmbi.ru.nl/cdd/biovenn/). For each time-points (1, 1.5, 2, and 4 h), the input gene lists were the list of DE genes for each NIL when expression was compared for the given time-point to expression at the 0 h control.

DE genes were identified within each NIL by comparing subsequent time-points to the 0 h control (Supplementary Table 3). In both NILs, the number of DE genes increased as the experiment progressed, and DE genes were located across all 12 chromosomes. In tolerant NIL175, DE genes increased from 371 at 1 h to 2677 at 4 h. In susceptible NIL163, DE genes increased from 228 at 1 h to 2238 at 4 h. In NIL163, only one DE gene was located within the introgressed region, and only at 4 h (Supplementary Table 3). Venn diagrams revealed overlap between the NILs for DE genes when time-point DE gene lists were compared (Figure 1). A majority of DE genes in NIL175 were shared with NIL163 only at 1.5 and 4 h. In contrast, the majority of DE genes for NIL163 were also DE in NIL175 for all time-points. NIL175 also had more DE genes than NIL163 at all time-points except at 1.5 h (Figure 1).

### GO enrichment of DE genes in NIL to NIL and time-point to time-point comparisons

GO enrichment analysis for the unique and overlapping gene lists from the time-point to time-point comparisons between the NILs identified significantly enriched GO terms for the majority of gene lists. The best by parent grouping and the full results of GO term enrichment are available in Table 1 and Supplementary Table 4. All overlapping gene lists from each time-point comparison had significantly enriched GO terms, which increased in number as the experiment progressed. The overlapping gene lists were predominantly enriched for GO terms related to protein modifications or transcriptional regulation. At 4 h, the comparison also showed enrichment for GO terms related to photosynthesis and plant response to light.

**Table 1:** **Consolidated GO term analysis of unique and overlapping gene lists from DE analysis of NIL175 and NIL163**.

**Time-Point (h)**	**NIL**	**GO term**	**Description**	**Functional Type**	**No. in Input List**	**No. in Reference**	**Corrected *P*-Value**
1	163	GO:0001071	Nucleic acid binding transcription factor activity	MF	11	699	0.017
1	Overlap	GO:0004402	Histone acetyltransferase activity	MF	3	12	0.00667
1	175	GO:0007154	Cell communication	BP	20	795	0.0157
1.5	Overlap	GO:0007154	Cell communication	BP	30	795	0.0107
1.5	Overlap	GO:0005992	Trehalose biosynthetic process	BP	5	19	0.00749
1.5	Overlap	GO:0001071	Nucleic acid binding transcription factor activity	MF	36	699	6.01E-07
1.5	Overlap	GO:0016407	Acetyltransferase activity	MF	8	68	0.00974
1.5	175	GO:0004674	Protein serine/threonine kinase activity	MF	15	515	0.0474
1.5	175	GO:0016740	Transferase activity	MF	50	3165	0.0237
1.5	175	GO:0043531	ADP binding	MF	12	252	0.00218
2	overlap	GO:0000160	Phosphorelay signal transduction system	BP	11	79	0.0455
2	Overlap	GO:0015698	Inorganic anion transport	BP	10	50	0.00372
2	Overlap	GO:0005991	Trehalose metabolic process	BP	7	20	0.00187
2	Overlap	GO:0043531	ADP binding	MF	29	252	3.99E-06
2	Overlap	GO:0004842	Ubiquitin-protein transferase activity	MF	17	143	0.00406
2	Overlap	GO:0004674	Protein serine/threonine kinase activity	MF	35	515	0.036
2	Overlap	GO:0047134	Protein-disulfide reductase activity	MF	7	21	0.00273
2	175	GO:0010167	Response to nitrate	BP	8	26	0.0161
2	175	GO:0006412	Translation	BP	89	536	2.79E-22
2	175	GO:0015706	Nitrate transport	BP	9	32	0.011
2	175	GO:0005840	Ribosome	CC	88	453	4.2E-27
2	175	GO:0004674	Protein serine/threonine kinase activity	MF	52	515	0.000286
2	175	GO:0030247	Polysaccharide binding	MF	11	48	0.0106
2	175	GO:0003735	Structural constituent of ribosome	MF	86	423	6.07E-28
2	175	GO:0043531	ADP binding	MF	41	252	4.96E-09
2	175	GO:0016597	Amino acid binding	MF	10	39	0.00899
4	163	GO:0009637	Response to blue light	BP	7	50	0.0463
4	163	GO:0000786	Nucleosome	CC	10	92	0.0125
4	Overlap	GO:0015979	Photosynthesis	BP	50	345	8.98E-08
4	Overlap	GO:1901700	Response to oxygen-containing compound	BP	48	486	0.0242
4	Overlap	GO:0007154	Cell communication	BP	70	795	0.02
4	Overlap	GO:0071495	Cellular response to endogenous stimulus	BP	28	209	0.00555
4	Overlap	GO:0006351	transcription, DNA-templated	BP	110	1341	0.00264
4	Overlap	GO:0009416	Response to light stimulus	BP	37	346	0.0379
4	Overlap	GO:0009521	Photosystem	CC	23	147	0.00271
4	Overlap	GO:0016407	Acetyltransferase activity	MF	14	68	0.00971
4	Overlap	GO:0016651	Oxidoreductase activity, acting on NAD(P)H	MF	19	106	0.0025
4	overlap	GO:0045156	Electron transporter, transferring electrons within the cyclic electron transport pathway of photosynthesis activity	MF	9	21	0.000477
4	overlap	GO:0001071	Nucleic acid binding transcription factor activity	MF	94	699	5.56E-14
4	overlap	GO:0043565	Sequence-specific DNA binding	MF	51	356	8.93E-08
4	175	GO:0031669	Cellular response to nutrient levels	BP	9	51	0.0446
4	175	GO:0006820	Anion transport	BP	15	134	0.0456
4	175	GO:0004674	Protein serine/threonine kinase activity	MF	48	515	1.92E-07
4	175	GO:0043531	ADP binding	MF	29	252	8.18E-06

*The lists of DE genes from the comparison of time-points 1, 1.5, 2, and 4 h to the 0 h control from each NIL were compared to produce input lists of unique or overlapping genes for GO enrichment analysis. GO term significance was determined at P ≤ 0.05. The following are given for each significant GO term: Time-point compared, NIL [either unique to 175 or 163, or overlapping (overlap)], the GO term returned, GO description, functional type (BP, biological process; MF, molecular function; CC, cellular component), the number of genes from the input list matching that GO term, the number of genes in the reference matching that GO term, and the corrected P-value. Results are sorted within time-point/NIL combination by functional type. S. lycopersicum cv. Heinz 1706 ITAGv2.40 was used as the reference. Only parent GO-terms are shown; for full GO results including all sub-terms please see Supplementary Table 2*.

Significantly enriched GO terms were identified for unique gene lists of NIL175 at all time-points. Similarly to the overlapping gene lists, enriched GO terms were related to protein modification and regulation. In addition, GO terms related to cell communication were identified at 1 h, and response to nutrient levels (specifically nitrate and nitrate transport) were identified at 2 h. NIL163 had significantly enriched GO terms in the unique gene lists only at 1 h (nucleic acid binding) and 4 h (nucleosome and response to blue light). Unique parent GO terms identified at 1 h for both NIL175 and NIL163 were significantly enriched in the list of overlapping genes at 1.5 h. Interestingly, tolerant NIL175 had enrichment of GO term GO:0004674 (protein serine/threonine kinase activity) starting at 1.5 h and through the end of the experiment. This GO term was also present in the overlapping gene list at 2 h, and was not represented in any other overlapping DE gene lists, or for any unique gene lists of NIL163 (Table 1, Supplementary Table 4). It is also interesting to note that no GO terms directly related to ABA signaling were significantly enriched, including those for membrane transport or signaling pathway regulation (Table 1, Supplementary Table 4).

### Clustering of expression patterns across time-points in NILs

Cluster analysis resulted in 48 unique expression pattern clusters for NIL175 and 146 unique clusters for NIL163. Cluster assignments for each gene are available for each NIL (see Supplementary Table 2). Visual examination revealed that clusters were based on both transcriptional pattern (i.e., changes in transcription across time-points), as well as general transcript level. For example, two genes with the same transcript expression pattern but a three-fold difference in relative level of transcription were sorted into different clusters. Cluster analysis of NIL175 and NIL163 did not show any evidence of genes that are expressed only under chilling stress conditions but not at the 0 h (non-stressed) control (Supplementary Table 2), as exemplified by the lack of clusters composed of genes with zero or near-zero expression levels at 0 h, followed by expression at subsequent time points as chilling stress was imposed.

**Figure 2:**
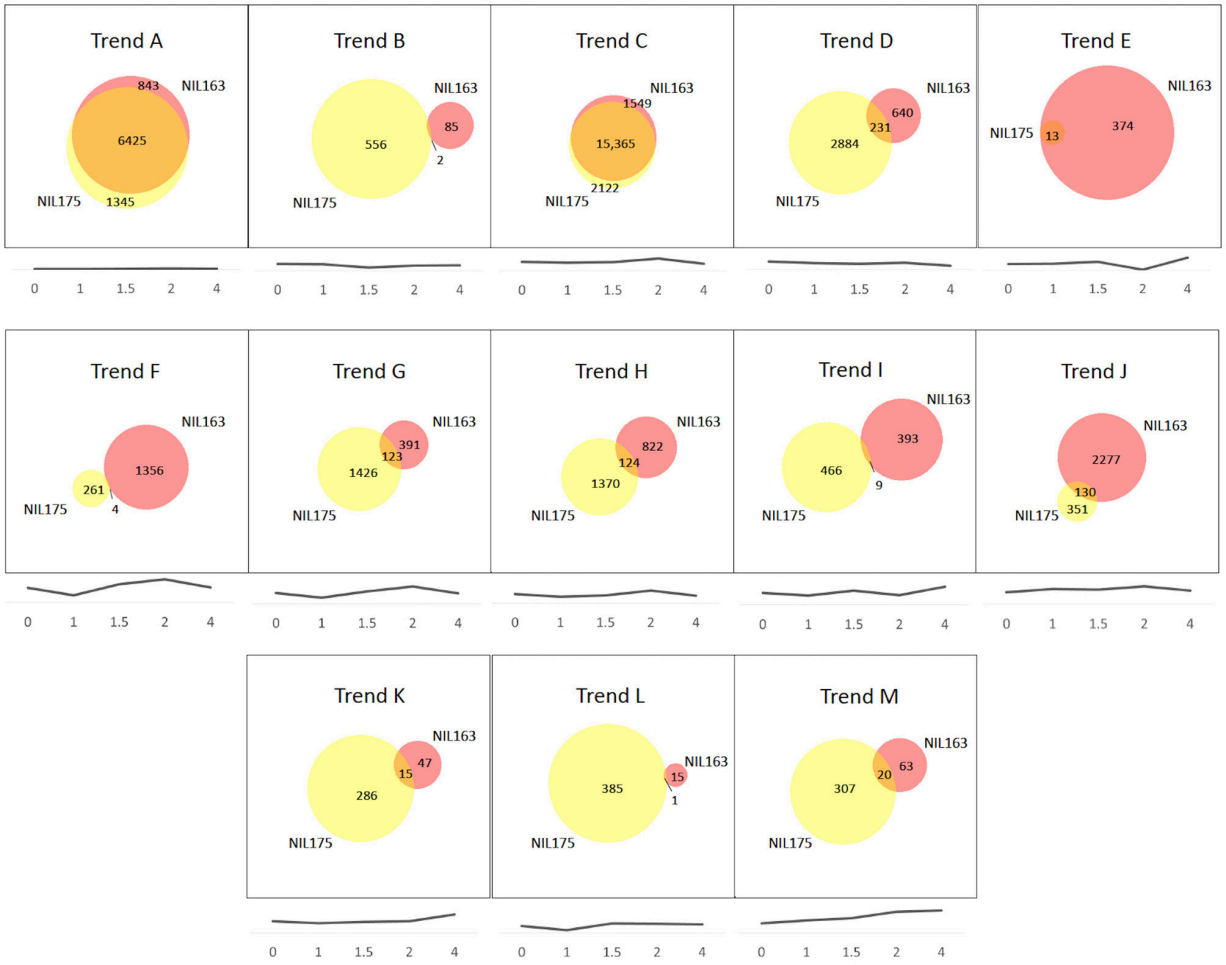
**Trend group comparisons of NIL175 and NIL163**. Trend Groups were identified from cluster analysis of gene transcription patterns for NIL175 and NIL163. For each Trend Group A-M, Venn diagrams show relative gene space of unique genes for NIL175 (yellow), NIL163 (pink), and overlapping genes (orange). Below each Venn diagram, the trend line corresponding to the given Trend Group is displayed. Experiment time-points in hours are designated along the x-axis. Y-axis values are in relative log scale (not shown), with y-axis range adjusted for each graph to maintain relative changes between time-points while displaying the trend line close to the x-axis for display purposes. Venn diagrams were created using BioVenn (www.cmbi.ru.nl/cdd/biovenn/).

Comparisons of Trend Groups revealed 13 were common to both NILs (designated Trend Groups A–M; Figure 2). In addition, NIL175 had one unique Trend Group (designated n), and NIL163 had eight unique Trend Groups (designated o–v). Figures showing each trend line are provided for A–M (see Figure 2) and n–v (see Supplementary Figure 1). For both NILs, Trend Groups A and C contained the largest number of genes, although the number of overlapping genes within each shared Trend Group (A–M) varied greatly (Figure 2). Trend Group A (no expression) and Trend Group C (very slight decrease to 1.5 h, peak at 2 h, and decrease at 4 h) had almost completely overlapping gene sets between the NILs. Trend Group E (increase to 1.5 h, decrease at 2 h, and highest level of expression at 4 h) contained only 13 genes in NIL175, all of which were represented in the larger gene list for NIL163 (Figure 1, Supplementary Table 2). For most Trend Groups, NIL175 had a larger set of genes than NIL163, with the exception of Trend Group E (mentioned previously), and Trend Group J (peaks at 1 and 2 h).

### GO enrichment in trend group comparisons of NIL175 and NIL163

All Trend Groups (with the exception of Trend Groups B and L) had significantly enriched GO terms in at least one of the three gene lists (unique to NIL175 or to NIL163 or overlapping). The best by parent grouping GO term analyses are given in Table 2. Full results of GO term enrichment are provided in an additional table (see Supplementary Table 5). The number of significantly enriched GO terms mirrored the number of genes in a list, with more populous gene lists containing more significantly enriched GO terms. Trend Group A was enriched for GO terms relating to DNA metabolism and transcription, including the transcriptional complex Mediator (GO:0016592), and DNA helicase (GO:0003678). Trend Group C was enriched for GO terms related to transcription factor regulation, protein modification, and transport. Trend Group E was unique in that it was the only Trend Group for which significantly enriched GO terms were identified for the unique set of genes in NIL163, but not the overlapping list or unique list for NIL175 (Table 2, Supplementary Table 5).

**Table 2:** **Consolidated GO term analysis of unique and overlapping genes from Trend Groups A-M identified by cluster analysis of NIL175 and NIL163**.

**Trend Group**	**NIL**	**GO term**	**Description**	**Functional Type**	**No. in Input List**	**No. in Reference**	**Corrected *p*-Value**
A	163		No significant results				
A	Overlap	GO:0000723	Telomere maintenance	BP	42	89	3.76E-17
A	Overlap	GO:0015074	DNA integration	BP	14	39	0.00655
A	Overlap	GO:0000785	Chromatin	CC	27	117	0.00993
A	Overlap	GO:0016592	Mediator complex	CC	13	37	0.0182
A	Overlap	GO:0004161	Dimethylallyltranstransferase activity	MF	19	25	3.26E-12
A	Overlap	GO:0032296	Double-stranded RNA-specific ribonuclease activity	MF	32	52	2.71E-17
A	Overlap	GO:0001076	RNA polymerase II transcription factor binding transcription factor activity	MF	13	27	0.000254
A	Overlap	GO:0030599	Pectinesterase activity	MF	28	125	0.0125
A	Overlap	GO:0008146	Sulfotransferase activity	MF	18	40	4.98E-06
A	Overlap	GO:0003674	Molecular function	MF	1778	16983	7.39E-26
A	Overlap	GO:0008234	Cysteine-type peptidase activity	MF	118	247	4.66E-52
A	Overlap	GO:0008270	Zinc ion binding	MF	209	1344	2.14E-10
A	Overlap	GO:0003678	DNA helicase activity	MF	43	85	4.12E-19
A	Overlap	GO:0008171	O-methyltransferase activity	MF	20	55	5.79E-05
A	Overlap	GO:0003676	Nucleic acid binding	MF	449	3222	3.32E-16
A	175	GO:0008234	Cysteine-type peptidase activity	MF	20	247	0.0253
A	175	GO:0046914	Transition metal ion binding	MF	83	1897	0.0461
B	163		No significant results				
B	Overlap		No significant results				
B	175		No significant results				
C	163	GO:0046274	Lignin catabolic process	BP	7	22	0.0262
C	163	GO:0009538	Photosystem I reaction center	CC	6	10	0.00113
C	163	GO:0052716	Hydroquinone:oxygen oxidoreductase activity	MF	7	22	0.0262
C	Overlap	GO:0006289	Nucleotide-excision repair	BP	18	18	0.0241
C	Overlap	GO:0009926	Auxin polar transport	BP	26	28	0.0195
C	Overlap	GO:0006366	Transcription from RNA polymerase II promoter	BP	35	38	0.00111
C	Overlap	GO:0044699	Single-organism process	BP	3849	5520	3.9E-120
C	Overlap	GO:0006354	DNA-templated transcription, elongation	BP	37	40	0.000411
C	Overlap	GO:0030964	NADH dehydrogenase complex	CC	21	22	0.0439
C	Overlap	GO:0005737	Cytoplasm	CC	2363	3079	3.2E-139
C	Overlap	GO:0004702	Receptor signaling protein serine/threonine kinase activity	MF	21	22	0.0439
C	Overlap	GO:0042623	ATPase activity, coupled	MF	139	186	0.000112
C	Overlap	GO:0016817	Hydrolase activity, acting on acid anhydrides	MF	487	716	5.37E-08
C	Overlap	GO:0016874	Ligase activity	MF	221	294	1.04E-08
C	Overlap	GO:0016853	Isomerase activity	MF	156	219	0.00301
C	Overlap	GO:0016772	Transferase activity, transferring phosphorus-containing groups	MF	1000	1633	0.0178
C	Overlap	GO:0016417	S-acyltransferase activity	MF	28	31	0.0335
C	Overlap	GO:0005198	Structural molecule activity	MF	352	481	8.62E-12
C	Overlap	GO:0019205	Nucleobase-containing compound kinase activity	MF	35	39	0.00491
C	Overlap	GO:0035639	Purine ribonucleoside triphosphate binding	MF	1627	2526	1.65E-15
C	Overlap	GO:0008565	Protein transporter activity	MF	32	33	0.000119
C	Overlap	GO:0004721	Phosphoprotein phosphatase activity	MF	79	102	0.00533
C	Overlap	GO:0003723	RNA binding	MF	408	567	5.92E-12
C	175	GO:0010207	Photosystem ii assembly	BP	18	98	0.0371
C	175	GO:0004674	Protein serine/threonine kinase activity	MF	59	515	0.00762
D	163		No significant results				
D	Overlap		No significant results				
D	175	GO:0003674	Molecular_function	MF	1271	16983	4.22E-11
D	175	GO:0016705	Oxidoreductase activity, acting on paired donors, with incorporation or reduction of molecular oxygen	MF	59	496	0.0276
D	175	GO:0043531	ADP binding	MF	46	252	1.03E-06
E	163	GO:0015979	Photosynthesis	BP	40	345	1.36E-29
E	163	GO:0009521	Photosystem	CC	27	147	1.02E-24
E	163	GO:0009507	Chloroplast	CC	22	942	0.0397
E	163	GO:0048038	Quinone binding	MF	6	41	0.00128
E	163	GO:0003735	Structural constituent of ribosome	MF	22	423	4.12E-08
E	163	GO:0016651	Oxidoreductase activity, acting on NAD(P)H	MF	16	106	1.7E-12
E	163	GO:0003899	DNA-directed RNA polymerase activity	MF	16	85	4.28E-14
E	163	GO:0019843	rRNA binding	MF	11	62	6.78E-09
E	Overlap		No significant results				
E	175		No significant results				
F	163		No significant results				
F	Overlap		No significant results				
F	175	GO:0009808	Lignin metabolic process	BP	10	29	7.27E-11
F	175	GO:0008150	Biological_process	BP	128	9489	0.000229
F	175	GO:0009834	Plant-type secondary cell wall biogenesis	BP	5	23	0.00167
F	175	GO:0015979	Photosynthesis	BP	24	345	3.66E-11
F	175	GO:0018298	Protein-chromophore linkage	BP	8	47	0.000011
F	175	GO:0010413	Glucuronoxylan metabolic process	BP	8	55	0.00004
F	175	GO:0006979	Response to oxidative stress	BP	11	258	0.0332
F	175	GO:0005576	Extracellular region	CC	24	339	2.49E-11
F	175	GO:0009521	Photosystem	CC	20	147	1.29E-14
F	175	GO:0046906	Tetrapyrrole binding	MF	26	494	1.94E-09
F	175	GO:0046872	Metal ion binding	MF	58	3123	0.000341
F	175	GO:0005507	Copper ion binding	MF	10	119	0.00018
F	175	GO:0016679	Oxidoreductase activity, acting on diphenols and related substances as donors	MF	10	41	3.67E-09
G	163		No significant results				
G	Overlap		No significant results				
G	175	GO:0009535	Chloroplast thylakoid membrane	CC	21	152	0.00111
G	175	GO:0046906	Tetrapyrrole binding	MF	40	494	0.0392
G	175	GO:0016491	Oxidoreductase activity	MF	107	1757	0.029
H	163	GO:0005515	Protein binding	MF	76	1739	0.000265
H	Overlap		No significant results				
H	175	GO:0044436	Thylakoid part	CC	30	301	0.00096
H	175	GO:0005515	Protein binding	MF	99	1739	0.0113
H	175	GO:0016705	Oxidoreductase activity, acting on paired donors, with incorporation or reduction of molecular oxygen	MF	37	496	0.0481
I	163		No significant results				
I	Overlap	GO:0009522	Photosystem i	CC	2	88	0.0278
I	175	GO:0018298	Protein-chromophore linkage	BP	6	47	0.00885
I	175	GO:0015979	Photosynthesis	BP	51	345	1.15E-39
I	175	GO:0009521	Photosystem	CC	37	147	4.91E-37
I	175	GO:0009055	Electron carrier activity	MF	23	213	1.65E-13
I	175	GO:0019843	rRNA binding	MF	12	62	2.43E-09
I	175	GO:0003735	Structural constituent of ribosome	MF	29	423	3.53E-12
I	175	GO:0048038	Quinone binding	MF	7	41	0.000213
I	175	GO:0016168	Chlorophyll binding	MF	8	49	0.000043
I	175	GO:0003899	DNA-directed RNA polymerase activity	MF	22	85	1.76E-21
J	163	GO:0009765	Photosynthesis, light harvesting	BP	11	41	0.019
J	163	GO:0009523	Photosystem II	CC	14	68	0.0394
J	Overlap		No significant results				
J	175	GO:0001071	Nucleic acid binding transcription factor activity	MF	18	699	0.0479
K	163		No significant results				
K	Overlap		No significant results				
K	175	GO:0022900	Electron transport chain	BP	8	119	0.00304
L	163		No significant results				
L	Overlap		No significant results				
L	175		No significant results				
M	163	GO:0015979	Photosynthesis	BP	8	345	0.000215
M	163	GO:0022900	Electron transport chain	BP	5	119	0.00236
M	163	GO:0032991	Macromolecular complex	CC	13	1694	0.00963
M	163	GO:0009521	Photosystem	CC	8	147	2.78E-07
M	Overlap		No significant results				
M	175	GO:0071365	Cellular response to auxin stimulus	BP	6	58	0.0248
M	175	GO:0048046	Apoplast	CC	8	119	0.0296
M	175	GO:0016831	Carboxy-lyase activity	MF	7	74	0.00989
M	175	GO:0008080	N-acetyltransferase activity	MF	6	60	0.0301
M	175	GO:0030170	Pyridoxal phosphate binding	MF	10	144	0.00241
M	175	GO:0003700	Sequence-specific DNA binding transcription factor activity	MF	26	699	2.28E-05
M	175	GO:0016762	Xyloglucan:xyloglucosyl transferase activity	MF	6	37	0.00171

*For each Trend Group, GO enrichment analysis of the genes unique to each NIL, as well as overlapping between the two NILs, was performed. GO term significance was determined at P ≤ 0.05. The following are given for each significant GO term: Trend Group (A-M), NIL [either unique to 175 or 163, or overlapping (overlap)], the GO term returned, GO description, functional type (BP, biological process; MF, molecular function; CC, cellular component), the number of genes from the input list matching that GO term, the number of genes in the reference matching that GO term, and the corrected P-value. Results are sorted within time-point/NIL combination by functional type. S. lycopersicum cv. Heinz 1706 ITAGv2.40 was used as the reference. Only parent GO-terms are shown; for full GO results including all sub-terms please see Supplementary Table 3*.

## Discussion

### GO terms significantly enriched in NIL175 and NIL163 in response to root chilling

Multiple gene networks have been identified in crop plant responses to abiotic stresses, including changes in stress signaling, protein metabolism, energy metabolism, ROS scavenging enzymes, and photosynthetic enzymes (Allen and Ort, 2001; Des Marais and Juenger, 2010; Kosová et al., 2015). In our study, we looked at genome-wide patterns of transcriptional regulation in a set of paired NILs that differed in their tolerance to rapid-onset water stress induced by root chilling. We observed overlap between root chilling-tolerant NIL175 and chilling susceptible NIL163 for significantly enriched GO terms related to cell communication, transcription factor activity, various metabolic processes, response to light, and response to oxygen-containing compounds. In gene lists unique to NIL175, significantly enriched GO terms were detected for cell communication, protein modification, and response to nutrient levels, while in NIL163 many of the significantly enriched GO terms were related to photosynthesis (Tables 1, 2, Supplementary Table 4). Although there are few published studies on transcriptional regulation in tolerant and susceptible tomato lines exposed to chilling stress, our results were generally consistent with, but also distinct from, other studies. Liu et al. (2012) compared gene expression in *S. lycopersicum* acc. LA4024 with wild *S. habrochaites* acc. LA1777 and LA3969 (a cultivated tomato line containing an introgression from *S. habrochaites* on chromosome 12). Whole plants were exposed to chilling stress at 4°C, and leaf samples were collected each day for 7 days. The authors reported that GO terms related to response to stimulus, response to stress, and metabolism were significantly enriched across all three lines. Metabolism and stress response-related GO terms were significantly enriched in tolerant LA1777 and LA3969, while in susceptible LA4024, defense response and circadian rhythm terms were significantly enriched (Liu et al., 2012). In a study on a chilling-tolerant tomato species, *S. lycopersicoides*, Chen et al. (2015a) identified GO term enrichment for response to stimulus, signaling, and cell death in leaves after whole plant exposure to chilling stress at 4°C compared to non-stressed controls. GO enrichment analysis provides a translation of a gene list into a functional profile of biological processes, cell components, and molecular functions overrepresented in the gene list. It may be negatively affected by the quality of the genome annotation, as well as the quality of the GO term annotation and association (Khatri and Draghici, 2005; Reimand et al., 2007; Gene Ontology Consortium et al., 2013). Therefore, it is possible that additional biological, cellular, and molecular processes involved in the root response to chilling-stress are not captured in this analysis.

An interesting discovery in our analysis was the lack of clusters in either NIL that showed evidence of transcriptional activation in response to root chilling (Table 2, Supplementary Table 4 and Supplementary Figure 1), and the significant enrichment of GO terms related to Mediator and DNA helicase in Trend Group A (no expression) for both NILs (Table 2, Supplementary Figure 1). Both Mediator and DNA helicase are essential for transcriptional activation of genes that are not already primed for transcription in eukaryotes, including plants (Lohman and Bjornson, 1996; Allen and Ort, 2001; Patel and Donmez, 2006; Wu and Nacu, 2010; Hentges, 2011; Wu, 2012; Poss et al., 2013). Our findings suggest that while de-novo transcription may be occurring, the response to chilling stress in roots may be more dependent on transcriptional regulation of genes already primed for expression, as well as post-transcriptional/post-translational modifications of transcripts or proteins already present in root cells. The importance of post-transcriptional and post-translational regulation of proteins in regulating transcriptional changes caused by osmotic stress has been reported previously and was recently reviewed by Guerra et al. (2015).

### The role of serine/threonine kinase activity in the response of NIL175 to root chilling

When plants are exposed to osmotic stress, a major mechanism of stress signaling within cells is increased enzymatic activity of kinases, referred to as osmotic stress-activated kinases (Kulik et al., 2011; Fujii and Zhu, 2012). The phytohormone abscisic acid (ABA) has been identified as fulfilling a critical role in coordinating plant response to osmotic stress in conjunction with various kinases and other regulatory molecules (Fujita et al., 2013). The serine/threonine kinase type 2 (SnRK2) family of kinases (plant equivalents of the Snf1 kinases in yeast) are predominantly involved in the cellular response to osmotic stress in both an ABA-dependent and ABA-independent manner (Coello et al., 2011; Yunta et al., 2011). SnRK2 kinases regulate ABA-responsive elements (ABRE) such as ABRE-binding proteins and ABRE-binding factors, transcription factors that regulate the ABRE-mediated regulation of downstream targets. Members of the SnRK2 family also have been shown to control stomatal closure in response to osmotic stress (Yunta et al., 2011; Fujita et al., 2013). The significant enrichment of protein serine/threonine kinase activity (GO term GO:0004647) in tolerant NIL175 in response to root chilling stress, but not in susceptible NIL163 (except for the overlapping gene set at 2 h), suggests that the SnRK2 kinase pathway plays a significant role in the NIL175 response to root chilling. This is further supported by the significant enrichment of GO:0004647 in only the unique gene list for NIL175 for Trend Group C in the Trend Group analyses. As our analyses did not show evidence of enrichment for ABA-specific GO terms, it is possible that the signaling of root-chilling stress in the roots occurs in an ABA-independent manner. These results do not preclude the involvement of ABA in the shoot response to root chilling stress, which was not investigated in these experiments.

The enrichment of protein serine/threonine kinase activity (GO:0004647) is also interesting in the context of the significant enrichment in NIL175 of GO terms related to cell communication at 1 h and response to nutrient levels at 2 and 4 h. Two other classes of SnRK kinases (SnRK1 and SnRK3) have been implicated in stress signaling in addition to more primary roles in cellular metabolism, including carbon, nitrogen, sucrose, and lipid metabolism (Laurie and Halford, 2001; Hey et al., 2010; Kulik et al., 2011). It is possible that SnRK kinases and their associated pathways may be serving multiple functions during plant exposure to root chilling, initially serving as part of the signaling cascade sensing and responding to root chilling stress, and eventually as modulators of metabolism throughout the duration of the stress episode.

### Root chilling stress causes distinct, But overlapping, transcriptional responses in NIL175 and NIL163

Our previous research documented that the physiological response to rapid-onset water stress induced by root chilling causes distinct phenotypic responses in susceptible and tolerant tomato lines that differ for the presence or absence of a *S. habrochaites* introgression on the short arm of chromosome 9 (Truco et al., 2000; Bloom et al., 2004; Goodstal et al., 2005; Easlon et al., 2013; Arms et al., 2015). In this study we discovered that while the roots of both NILs exhibited some overlap in their transcriptional response to root chilling, unique responses were also evident. Plant transcriptional responses to abiotic stresses are complex and overlapping, and a core set of stress response genes are rapidly induced in response to a range of stresses (Kültz, 2005; Walley and Dehesh, 2010). In general, plant responses to abiotic stresses can be divided into overlapping responses that are exhibited in response to a variety of stresses, vs. specific responses that result in re-establishment of homeostasis in a manner that is specific to the imposed stress (Kültz, 2005). Overlap observed in the DE time-point comparisons between the NILs as well as in Trend Groups A–M may represent a general response to stress that is common to multiple types of abiotic stress, and may also be shared across tomato species. Transcriptional patterns not shared by the two NILs may be the result of each NIL responding to root chilling in a genotype- and stress-specific manner.

A pattern of overlapping and unique transcriptional responses has also been observed in other studies of tolerant and susceptible tomato genotypes exposed to chilling or drought stress (Gong et al., 2010; Chen et al., 2015b). Chen et al. (2015b) compared transcriptional regulation in leaves of chilling-tolerant *S. habrochaites* LA1777 to sensitive *S. lycopersicum* cv. Glamour exposed to 4°C for 0, 1, and 12 h. The majority of gene regulation patterns were dissimilar, yet some genes showed overlapping patterns in both susceptible and tolerant genotypes. Gong et al. (2010) reported similar transcriptional patterns as Chen et al. (2015b) when comparing *S. lycopersicum* cv. M82 with tomato lines containing *S. pennellii* introgressions on chromosome 2 (line IL2-5) and chromosome 9 (line IL9-1, with an introgression syntenic to the introgression in our NIL175) exposed to drought stress for several days. The findings of these two studies are in parallel with our observations of a combination of unique and overlapping transcriptional patterns in our two NILs. Furthermore, these studies compared a tomato cultivar to either a wild species or to cultivated tomato containing a wild species introgression, thus the observed overlapping transcriptional responses imply that cultivated tomato shares portions of general stress response pathways with its wild relatives but without exhibiting the same phenotypic tolerances to some abiotic stressors.

### Transcriptional response to root chilling in NIL175 is more targeted, yet Is more complex in NIL163

We had hypothesized that chilling-tolerant NIL175 would exhibit a more complex transcriptional response to root chilling in comparison to susceptible NIL163, however a larger number of clusters was identified for NIL163 than for NIL175 (Supplementary Table 2). This implies greater transcriptional complexity in chilling-sensitive NIL163, while the greater enrichment of significant GO terms (general and stress-specific) in unique gene lists for NIL175 in both the time-point comparisons for DE genes as well as for the Trend Groups imply a more focused transcriptional response to root chilling stress in NIL175 compared to NIL163 (Tables 1, 2, Supplementary Table 4 and Supplementary Figure 1). Significantly enriched GO terms represent an over-enrichment of genes with similar functions or within the same network for a given input gene list (Khatri and Draghici, 2005; Reimand et al., 2007). A similar result was reported by Gong et al. (2010) in that drought-susceptible *S. lycopersicum* cv. M82 exhibited a larger number of DE genes in leaves in response to drought stress than did tolerant tomato lines containing *S. pennellii* introgressions. Interestingly, although M82 showed a larger number of DE genes, the tolerant lines had a higher number of stress-responsive transcription factors that were differentially expressed. This result is similar to our findings that susceptible NIL163 had a more complex pattern of transcriptional regulation, and tolerant NIL175 exhibited a more targeted transcriptional response to root chilling stress. Research by Liu et al. (2012) on chilling stress in tomato leaves also showed a more focused transcriptional response in tolerant wild *S. habrochaites* LA1777 and in LA3696, a tomato line containing an *S. habrochaites* introgression in comparison to susceptible *S. lycopersicum* LA4204 after whole plant exposure to 4°C.

### Effect of *S. habrochaites* chromosome 9 introgression on global transcription under non-stress conditions

Of the 34,725 annotated genes in the *S. lycopersicum* cv. Heinz 1706 reference genome ITAG v2.40 (SGN, solgenomics.net), only 20 genes were differentially transcribed between our paired NILs at the non-stressed 0 h control (Supplementary Table 3). The very small number of DE genes in the non-stressed control suggests that there was little effect of the *S. habrochaites* chromosome 9 introgression on global transcription under non-stress conditions in our experiment. Interestingly, the majority of the 20 DE genes at 0 h were located on chromosome 9, although outside of the introgressed region. Previous work in *Arabidopsis thaliana* and *Thellungiella salsuginea* indicates that tolerant genotypes tend to exhibit increased levels of stress-inducible and stress-responsive proteins even in the absence of stress (Pang et al., 2010; Wendelboe-Nelson and Morris, 2012; Kosová et al., 2015). This suggests that the *S. habrochaites* introgression present in NIL175 may lead to a priming of the abiotic stress response pathways involved in root chilling tolerance, and that many of these response genes appear to be located on chromosome 9. Since there was an absence of enriched GO terms for DE genes between the two NILs at 0 h, it is not possible to interpret whether specific functional gene groups or networks were preferentially targeted. This evidence, along with the results discussed under “*GO terms significantly enriched in NIL175 and NIL163 in response to root chilling”* (above) suggests that while the chromosome 9 introgression from *S. habrochaites* may prime NIL175 to respond to root chilling stress in a tolerant manner in our experiment, it did not fundamentally alter transcription of specific functional gene networks, or genome-wide transcription levels.

## Author contributions

EA and DS conceived of and designed the experiment. EA and ZY conducted the experiment, collected biological samples, extracted RNA, prepared sequencing libraries and obtained Illumina sequence data. EA and ZY performed statistical and bioinformatics data analyses. EA and DS prepared and wrote the manuscript. All authors read and approved the final manuscript.

### Conflict of interest statement

The authors declare that the research was conducted in the absence of any commercial or financial relationships that could be construed as a potential conflict of interest.
