# Supplementary material for: Differential Transcriptional Regulation in Roots of Tomato Near-Isogenic Lines in Response to Rapid-Onset Water Stress
Source: Front Plant Sci. 2017 Feb 21;8:166. doi: 10.3389/fpls.2017.00166 (PMC5318454; doi:10.3389/fpls.2017.00166)

Supplementary Figure 1. Trend lines unique to NIL175 (n), and NIL163 (o-v). The trend line corresponding to the given Trend Group is displayed. Experiment time-points are designated along the x-axis. Y-axis values are in relative log scale (not shown), with y-axis range adjusted for each graph to maintain relative changes between time-points while displaying the trend line close to the x-axis for display purposes.

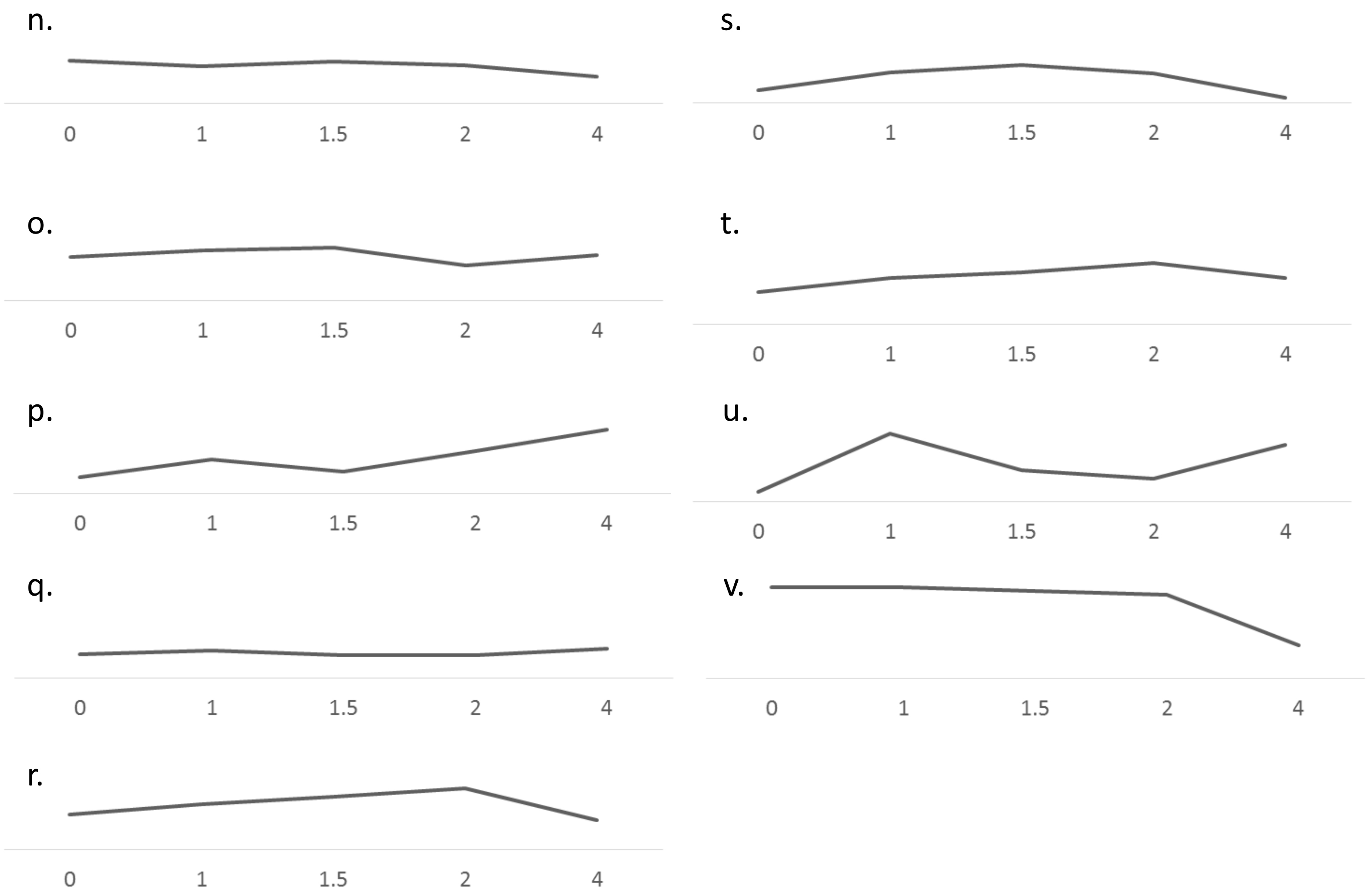

Supplement: Supplementary file 6 [file Image1.pdf]
